# Rheumatologists’ ultrasound confidence and technique are improved by a two day cadaveric sonoanatomy course

**DOI:** 10.1186/1471-2474-14-S1-A13

**Published:** 2013-02-14

**Authors:** Iain Goff, David Wright, Debra Patten

**Affiliations:** 1 Rheumatology, Newcastle Hospitals NHS Foundation Trust, Newcastle upon Tyne, NE7 7DN, UK; 2 Institute of Cellular Medicine, Newcastle University, Newcastle upon Tyne, UK; 3 Rheumatology, Sunderland Royal Hospital, Sunderland, SR4 7TP, UK; 4 Anatomy Department, Newcastle University, Newcastle upon Tyne, NE2 4HH, UK

## Background

Correct interpretation of musculoskeletal ultrasound (MSUS) requires thorough knowledge of normal anatomy, but several authors report deficiencies in anatomy skills among rheumatologists. Cadaver-based anatomy review courses improve clinical and injection skills, but the value of such courses in MSUS training is unclear. During 2010-12 we delivered two cadaver based, MSUS anatomy courses for the British Society of Rheumatology (BSR), and measured confidence to perform key MSUS learning objectives before and after the course using a self-assessment questionnaire.

## Methods

The two day course in March 2012 consisted of orientation lectures with MR imaging; expert led, small group workshops handling cadaveric specimens; simultaneous access to real-time ultrasound on live models; and ultrasound practice on patients with pathological anatomy. Ten item confidence logs based on BSR core competency outcomes and ability to diagnose EULAR pathologies were completed by delegates pre-course, end of course and four weeks post course. Standardised imaging protocols with anatomy checklists devised by tutors from the BSR ultrasound special interest group were used to guide scanning technique and to assess delegate ability to locate specific anatomic structures with ultrasound.

## Results

Twenty delegates attended the course. Delegate feedback rated the course very highly (Overall mean satisfaction score = 4.25, 1=poor, 5=excellent). Confidence logs collected from all 20 delegates demonstrated low levels of confidence in core domains pre-course (mean 3.5/10), improving to mean 5.5/10 immediately post-course (paired t-test p< 0.001). Confidence was maintained at 4 weeks in 7/10 domains (paired t-test p<0.05).

Anatomy checklists were completed and returned by 13 delegates. Delegates were able to identify most structures at the shoulder, elbow, wrist, knee and ankle (68-72% of structures located) though fewer structures were visualised at the hip (44%).

## Conclusion

The educational model used led to significant improvements in delegate’s confidence in identifying anatomical structures using MSUS, which were maintained at 4 weeks post course. Expert led, small group workshops handling cadaveric specimens with simultaneous practice of MSUS on live models is an effective model for MSUS anatomy training. This method of teaching was highly regarded by the delegates, and imaging protocols and checklists are a useful tool for self assessment.

